# Applying machine learning techniques to the identification of late-onset hypogonadism in elderly men

**DOI:** 10.1186/s40064-016-2531-8

**Published:** 2016-06-16

**Authors:** Ti Lu, Ya-Han Hu, Chih-Fong Tsai, Shih-Ping Liu, Pei-Ling Chen

**Affiliations:** Department of Psychiatry, Kaohsiung Veterans General Hospital, Kaohsiung, Taiwan, ROC; Department of Information Management, Institute of Healthcare Information Management, National Chung Cheng University, Chiayi, 621 Taiwan, ROC; Department of Information Management, National Central University, Jhongli, 320 Taiwan, ROC; Department of Urology, National Taiwan University Hospital, Taipei, 100 Taiwan, ROC

**Keywords:** Late-onset hypogonadism (LOH), Data mining, Classification, Prediction

## Abstract

In the diagnosis of late-onset hypogonadism (LOH), the Androgen Deficiency in the Aging Male (ADAM) questionnaire or Aging Males’ Symptoms (AMS) scale can be used to assess related symptoms. Subsequently, blood tests are used to measure serum testosterone levels. However, results obtained using ADAM and AMS have revealed no significant correlations between ADAM and AMS scores and LOH, and the rate of misclassification is high. Recently, many studies have reported significant associations between clinical conditions such as the metabolic syndrome, obesity, lower urinary tract symptoms, and LOH. In this study, we sampled 772 clinical cases of men who completed both a health checkup and two questionnaires (ADAM and AMS). The data were obtained from the largest medical center in Taiwan. Two well-known classification techniques, the decision tree (DT) and logistic regression, were used to construct LOH prediction models on the basis of the aforementioned features. The results indicate that although the sensitivity of ADAM is the highest (0.878), it has the lowest specificity (0.099), which implies that ADAM overestimates LOH occurrence. In addition, DT combined with the AdaBoost technique (AdaBoost DT) has the second highest sensitivity (0.861) and specificity (0.842), resulting in having the best accuracy (0.851) among all classifiers. AdaBoost DT can provide robust predictions that will aid clinical decisions and can help medical staff in accurately assessing the possibilities of LOH occurrence.

## Background

With the advent of an aging society, the population of elderly people gradually increases. Therefore, menopause-related problems are a prime concern worldwide. In elderly women, the incidence of diseases is higher after menopause and if such clinical conditions are ignored, they damage quality of life, interfere with organ function, and may even increase the risk of fatal diseases.

Late-onset hypogonadism (LOH), commonly known as andropause, is a clinical and biochemical syndrome that is mainly caused by the gradual decrease in testosterone levels in men. Patients with LOH typically exhibit aging with a low serum testosterone level (Araujo et al. 2004; Jones 2009, 2010). LOH-related symptoms are associated with a decrease in total testosterone (TT) levels, cessation of the secretion rhythm, increase in sex hormone-binding globulin (SHBG) levels, and decline in bioavailable free testosterone (FT) levels (Wang et al. 2008).

According to a study in the United States, in 2006, up to 600 million US dollars were spent on testosterone medication for the treatment of andropause, and other diseases related to low testosterone levels in andropause were not included. The results of an academic survey by the Taiwan Male Medical Association revealed that up to one-third of Taiwanese men aged over 40 years had androgen deficiency (based on blood sampling tests). Previous studies have revealed that because of the lack of andropause management, the life expectancy of men was shorter than that of women (Jones 2009, 2010).

In recent years, an increasing number of scholars have focused on andropause-related problems (Wang et al. 2008; Clapauch et al. 2008; Cunningham 2006; Gooren 2008; Haren et al. 2005; Iwamoto et al. 2009; Kalinchenko et al. 2008; Karazindiyanoğlu and Cayan 2008; Kalyani and Dobs 2007; La Vignera et al. 2008; Miner and Seftel 2007; Rosano et al. 2006; Wu et al. 2008, 2010). Compared with menopause, the occurrence of andropause is more inconsistent, and the symptoms of andropause are also relatively mild, which can be easily ignored. This results in a lack of timely and effective clinical assessments and hinders the prevention of LOH.

To diagnose LOH, the patient’s blood is sampled to measure the TT or FT levels. If the TT level is lower than 300 ng/dl or the FT level is lower than 6.5 ng/dl, then the patient has LOH; however, both TT and FT levels are not measured during routine inspections, and the tests to determine TT and FT levels are expensive. Few people actively undergo this test; therefore, many patients with LOH cannot be identified in a timely manner and are not treated. In clinical practice, the Androgen Deficiency in the Aging Male questionnaire (ADAM) or Aging Males’ Symptoms scale (AMS) are typically used to screen men suspected of LOH (Emmelot-Vonk et al. 2011; Heinemann et al. 1992; Heinemann 2005; Myon et al. 2009; Tancredi et al. 2004; Valenti et al. 2009). Based on the scores, if the patient’s assessments match the criteria of andropause symptoms, physicians perform blood sampling to confirm the presence of LOH. In other words, ADAM and AMS are methods that assist physicians in diagnosing andropause.

The ADAM and AMS are popular, simple, clinical self-assessment methods. In many studies, scientists have investigated the correlations between ADAM or AMS and the TT level. However, the results have shown no significant correlations between the methods and the levels, and the rate of misclassification is high (Wu et al. 2008, 2010; Emmelot-Vonk et al. 2011). Conversely, many studies in recent years have reported significant associations between clinical conditions such as the metabolic syndrome, obesity, and lower urinary tract symptoms (LUTS), and LOH (Kalinchenko et al. 2008; Karazindiyanoğlu and Cayan 2008; Kalyani and Dobs 2007; La Vignera et al. 2008). Moreover, the relevant test data can be easily obtained through routine inspections. If LOH can be determined by considering these factors, the accuracy of prediction can be increased, which will be helpful in reducing the probability of misclassification of LOH and the health care costs in clinical practice.

In this study, we sampled 772 clinical cases in cooperation with the physical examination center of a medical center in Northern Taiwan. The inclusion criterion for all cases in this study was the completion of the ADAM and AMS questionnaires. Two well-known classification techniques, the decision tree (DT) (Han et al. 2011; Liu et al. 2014; Quinlan 1987) and logistic regression (LGR), were used to construct the LOH prediction models on the basis of the aforementioned features. In addition, we also used the Adaptive Boosting (AdaBoost) technique to increase the accuracy of the model (Freund and Schapire 1997).

## Methods

### Data

With the help of National Taiwan University Hospital (NTUH) (the largest medical center in Taiwan), data from male patients who completed both the health checkup and questionnaires (ADAM and AMS) from October 2008 to November 2009 were obtained as the experimental dataset. The NTUH Institutional Review Board approved the study protocol (201207058RIC).

After referring to the relevant literature and discussing with physicians, the following metabolic syndrome indices were included as the input variables in this study: age, 4 LUTS indices (Qmax, Qmean, FT, and IPSS), AC sugar, systolic blood pressure (SBP), diastolic blood pressure (DBP), triglyceride (TG), high-density lipoprotein (HDL), and wrist. Based on patient history, the presence of hypertension was annotated for patients. In addition, glycated haemoglobin (HbA1c), body mass index (BMI), total cholesterol, PC sugar, obesity, diabetes, and other chronic disease-related information was included. Consequently, each data sample contained 16 input variables. Regarding the output variables, the free testosterone (FT) > 6.5 was defined as having no LOH symptoms and a score of FT ≤ 6.5 was defined as having LOH symptoms.

Consequently, raw data from the hospital included 1040 entries. As the baseline for this study, we also collected the ADAM and AMS assessment results simultaneously for all patients. After confirmation by experts and the deletion of data with missing values and outliers, 772 entries were retained for the research datasets to train and test the prediction models. Among these entries were 567 patients with no LOH symptoms and 205 patients with LOH symptoms. The descriptive statistics are shown in Table 1.

**Table 1:** Summary of input variables of clinical case and descriptive statistics

Input variable	Description	Range	Descriptive statistics
Age	Age of aging male (in years)	41–80	*μ* = 55.61; *σ* = 7.912
Qmax	Maximal flow rate (ml/s)	3–56	*μ* = 17.62; *σ* = 7.111
Qmean	Average flow rate (ml/s)	2–34	*μ* = 9.76; *σ* = 4.173
FT	Total flow time (s)	10–119	*μ* = 35.26; *σ* = 15.306
IPSS total	Total IPSS score	0–33	*μ* = 8.11; *σ* = 6.519
SBP	Systolic blood pressure (mmHg)	80–169	*μ* = 123.42; *σ* = 13.740
DBP	Diastolic blood pressure (mmHg)	43–111	*μ* = 73.77; *σ* = 9.845
HT	Hypertension	Yes or no	Yes: 315 (40.8 %) No: 457 (59.2 %)
AC sugar	AC Blood Sugar (mg/dl)	69–292	*μ* = 98.21; *σ* = 17.885
TG	Triglyceride (mg/dl)	31–676	*μ* = 133.44; *σ* = 71.750
HDL	High-density lipoprotein (mg/dl)	24–96	*μ* = 45.65; *σ* = 9.957
Wrist	Wrist (cm)	66.3–114.5	*μ* = 87.962; *σ* = 7.5566
HBA1c	Glycohemoglobin (%)	4.4–10.4	*μ* = 5.691; *σ* = 0.6133
BMI	Body mass index (mmHg)	16.7–36.7	*μ* = 24.807; *σ* = 2.8593
Total cholesterol	Total cholesterol (mg/dL)	95–440	*μ* = 203.18; *σ* = 34.138
PC sugar	PC blood sugar (mg/dl)	51–408	*μ* = 127.08; *σ* = 48.775
LOH		Y/N	Y: 205 (26.6 %) N: 567 (73.4 %)

### Investigated classification techniques

To construct LOH prediction models, we employed 2 well-known classification procedures, DT and LGR. The DT classifier is a well-known and powerful supervised learning technique with a hierarchical structure that comprises nodes and branches (Han et al. 2011; Liu et al. 2014; Quinlan 1987). In a DT, an internal node represents one of the independent variables, the branch of an internal node represents a subset of the values of the corresponding independent variable, and a leaf node is associated with a value (or a class label) of the dependent variable. The main advantage of using a DT is that the generated rules can be easily observed and interpreted, thus reducing the possibilities of mistakes in complex problems.

Many DT-based learning techniques have been developed in the past decades, and C4.5, proposed by Quinlan, is the most commonly used technique. The tree-generation process for C4.5 consists of the growing and pruning phases. The growing phase adopts a divide-and-conquer approach to select suitable variables in an internal node of DT and divides the training dataset into subsets by the selected attribute value. This process is recursively applied to each internal node (i.e., a subset of the training dataset) until any of the stop criteria are satisfied. At the same time, a class label is assigned to a leaf node based on majority voting. The pruning phase reduces the size of a DT to decrease the effect of noise on data and avoid over fitting. The prepruning approach is adopted by C4.5, which calculates the pessimistic error rate from the training dataset to decide when to stop growing a DT.

LGR is a widely used statistical procedure for modeling a dependent variable by a linear combination of 1 or more independent variables. The main difference between LGR and linear regression is that LGR deals with binomial or multinomial classification problems, whereas linear regression requires the dependent variable to be of interval or ratio scales. LGR aims to predict the occurrence probability of an event by fitting data into a logistic function, thereby allowing inputs with any values to be transformed and confined to values between 0 and 1.

The classifier ensemble technique was further employed to enhance the prediction power of the preceding 2 classification techniques. AdaBoost (Freund and Schapire 1997) is one of the most well-known classifier ensembles. AdaBoost iteratively applies a selected classification algorithm and evaluates each instance in the training dataset. For instances incorrectly classified by the current classifier, the misclassification cost increases for the next round of learning; in other words, AdaBoost encourages a new classifier to learn from instances misclassified by the earlier classifier by assigning a larger weight to those instances. After a sequence of classifiers is built, AdaBoost uses a weighted majority vote to make predictions. Although the concept of AdaBoost is simple, previous studies have shown that several classification algorithms in conjunction with AdaBoost achieve higher classification accuracy than individual base classifiers do.

### Experimental setup and performance measurement

The WEKA 3.6.4 open-source data mining software (www.cs.waikato.ac.nz/ml/weka) was employed to construct the LOH prediction model. Table 2 lists the specific parameter values selected for performing the C4.5, LGR, and AdaBoost classification techniques. In the collection dataset, only 35 % of men were diagnosed with LOH, which resulted in class imbalance. Because the adjustment of the ratio of the 2 class samples can improve a machine’s learning performance, we used the resampling method in WEKA to modify the distribution of instances of the 2 classes to be almost identical. In addition, some useful instances in the adequate class were not chosen by the resampling method, resulting in the loss of valuable information for classifications. Therefore, the random resampling method was applied 30 times to construct datasets. A tenfold cross-validation method was used in all the experimental evaluations. Because both AMS scale and ADAM questionnaire have been widely used in clinical practice for identifying LOH patients, we considered the evaluation results of these 2 methods as the baselines.

**Table 2:** Parameter settings in each classification technique

Technique	Parameters	Value
C4.5	BinarySplits	False
	ConfidenceFactor	0.25
	MinNumObj	2
	ReducedErrorPruning	False
LGR	HeuristicStop	50
	MaxBoostingIterations	500
	NumBoostingIterations	0
Adaboost	NumIterations	10
	WeightThreshold	100

To evaluate the performance of the constructed classification systems (i.e., prediction models), the accuracy, sensitivity, and specificity of each classifier was assessed. These were measured using a confusion matrix, as shown in Table 3.

**Table 3:** Confusion matrix

↓predicted/actual→	LOH symptoms	Non-LOH symptoms
LOH symptoms	True positive (a)	False positive (b)
Non-LOH symptoms	False negative (c)	True negative (d)

The average prediction accuracy (ACC), sensitivity, and specificity were obtained using the following formulas:

1 $${\text{Prediction}}\,{\text{accuracy}} = \frac{a + d}{a + b + c + d}$$

2 $${\text{Sensitivity}} = \frac{a}{a + c}$$

3 $${\text{Specificity}} = \frac{d}{b + d}.$$

## Results

The experimental results of each classifier and the baselines are shown in Table 4. Note that we generated thirty resampled datasets, resulting in generating thirty results for each experiment. All of the experimental evaluations reported in Table 4 are the averages of the results of thirty trials. In addition, the cross-validated paired *t* test was applied to compare performances of each pair of classifiers. The ACC values obtained using ADAM, AMS, DT, AdaBoost DT, LGR, and AdaBoost LGR, were 0.470, 0.478, 0.825, 0.851, 0.635, and 0.635, respectively. The results indicated that both ADAM and AMS have poor prediction performance in identifying LOH. Among all other classifiers, DT-based techniques significantly outperformed the LGR techniques at the 0.05 level (DT vs LGR: t = 43.268, p < 0.000; DT vs AdaBoost LGR: t = 44.106, p < 0.000; AdaBoost DT vs LGR: t = 51.875, p < 0.000; and AdaBoost DT vs AdaBoost LGR: t = 52.824, p < 0.000).

**Table 4:** Experimental results for each classifier

Method	ACC	Sensitivity	Specificity
ADAM	0.470	0.878	0.099
AMS	0.478	0.652	0.319
DT	0.825	0.840	0.812
AdaBoost DT	0.851	0.861	0.842
LGR	0.635	0.565	0.698
AdaBoost LGR	0.635	0.565	0.698

For the other 2 metrics, the sensitivities of ADAM, AMS, DT, AdaBoost DT, LGR, and AdaBoost LGR were 0.878, 0.652, 0.840, 0.861, 0.565, and 0.565, respectively. The sensitivities of ADAM, AMS, DT, AdaBoost DT, LGR, and AdaBoost LGR were 0.099, 0.319, 0.812, 0.842, 0.698, and 0.698, respectively. Although the sensitivity of ADAM is the highest, it has the lowest specificity, which implies that ADAM overestimates LOH occurrence. LGR and AdaBoost LGR tend to have higher specificities and lower sensitivities. Both DT and AdaBoost DT have relatively stable sensitivity and specificity. In addition, using the AdaBoost technique can improve the performance in DT, but not in LGR.

Because LOH-affecting factors are highly diverse, diagnosing LOH is difficult. Most studies on LOH have focused on identifying the relationships between LOH and other diseases. In this study, our aim was to develop a robust LOH prediction model for clinical use. According to the results mentioned in fourth section, neither ADAM nor AMS can provide satisfactory predictions for clinical use, and AdaBoost DT was the most accurate classifier in our study.

## Discussion

In DT classifiers, age was the most crucial variable and thus served as the first attribute to divide the patients into groups. Other critical variables included PC sugar, wrist, TG, HDL, and obesity, which are also used to split the internal nodes of DT. This means that the correlations between LOH and obesity or the metabolic syndrome were higher than those between LOH and LUTS. In AdaBoost DT classifiers, age remained the most critical variable, but metabolic syndrome-related variables (wrist, TG, HDL, hypertension, and AC sugar) had greater significance than the other variables. Diabetes-related variables (PC sugar and HBA1c) also appeared in the trees, showing that the metabolic syndrome, diabetes, and LOH had significant correlations.

The results of our study are consistent with those of previous studies that have explored the relationships between LOH and other diseases. Kalyani and Dobs (2007) investigated the relationships among testosterone deficiency syndrome (TDS), comorbidity of diabetes, and metabolic syndrome and found that 20–64 % of male patients with diabetes exhibited TDS, and it was more prevalent among elderly people. Testosterone deficiency may be a risk factor for diabetes and the metabolic syndrome. Conversely, the risk factors for diabetes and metabolic syndrome are similar to those for TDS. La Vignera et al. (2008) conducted a case study of 60 men (age, 54–63 years) with coexisting LOH and metabolic syndrome and found that a good supply of testosterone can alleviate the metabolic syndrome. Gooren (2008) found that TT concentration has a significant relationship with aging, particularly the metabolic syndrome.

## Conclusion

Because of the complexity of the psychological and physiological symptoms of LOH, developing an LOH prediction model has thus far been extremely difficult. In this study, we used retrospective data to construct an LOH predictive model, using various machine learning techniques and compared them with clinical LOH assessment methods. The results show that AdaBoost DT has the strongest performance and can be used in clinical practice. This study provides a stable clinical decision support system that helps clinicians in assessing the probability of occurrence of LOH. The system can provide instantaneous suggestions for a physician’s clinical judgment.

However, our study has limitations. First, the dataset was collected from a single medical center in Taiwan. The scope of this study can be further expanded to include samples from other hospitals. Second, this study only considered LOH-related physical symptoms. Other psychological and environmental factors that affect LOH occurrence can be considered in model development. Third, other machine learning techniques, such as support vector machines and bagging classifier ensembles, can be employed for further comparison.

## References

[CR1] Araujo AB, O’Donnell AB, Brambilla DJ, Simpson WB, Longcope C, Matsumoto AM (2004). Prevalence and incidence of androgen deficiency in middle-aged and older men: estimates from the Massachusetts Male Aging Study. J Clin Endocrinol Metab.

[CR2] Clapauch R, Carmo AM, Marinheiro L, Buksman S, Pessoa I (2008). Laboratory diagnosis of late-onset male hypogonadism andropause. Arq Bras Endocrinol Metabol: ABE&M.

[CR3] Cunningham GR (2006). Testosterone replacement therapy for late-onset hypogonadism. Nat Clin Pract Urol.

[CR4] Emmelot-Vonk MH, Verhaar HJJ, Nakhai-Pour HR, Grobbee DE, van der Schouw YT (2011). Low testosterone concentrations and the symptoms of testosterone deficiency according to the Androgen Deficiency in Ageing Males (ADAM) and Ageing Males’ Symptoms rating scale (AMS) questionnaires. Clin Endocrinol.

[CR5] Freund Y, Schapire RE (1997). A decision-theoretic generalization of on-line learning and an application to boosting. J Comput Syst Sci.

[CR6] Gooren L (2008). Can the administration of testosterone to men with late-onset hypogonadism be discontinued?. J Mens Health.

[CR7] Han J, Kamber M, Pei J (2012). Data mining: concepts and techniques.

[CR8] Haren M, Chapman I, Coates P, Morley J, Wittert G (2005). Effect of 12 month oral testosterone on testosterone deficiency symptoms in symptomatic elderly males with low-normal gonadal status. Age Ageing.

[CR9] Heinemann LAJ (2005). Aging Males’ Symptoms scale: a standardized instrument for the practice. J Endocrinol Invest.

[CR10] Heinemann LAJ, Zimmermann T, Vermeulen A, Thiel C, Hummel W (1992). A new aging males’ symptoms’ rating scale. Aging Male.

[CR11] Iwamoto T, Yanase T, Horie H, Namiki M, Okuyama A (2009). Late-onset hypogonadism (LOH) and androgens: validity of the measurement of free testosterone levels in the diagnostic criteria in Japan. Int J Urol.

[CR12] Jones TH (2009). Late onset hypogonadism. BMJ.

[CR13] Jones TH (2010). Andrology: identifying late-onset hypogonadism in older men. Nat Rev Urol.

[CR14] Kalinchenko S, Vishnevskiy EL, Koval AN, Mskhalaya GJ, Saad F (2008). Beneficial effects of testosterone administration on symptoms of the lower urinary tract in men with late-onset hypogonadism: a pilot study. Aging Male.

[CR15] Kalyani RR, Dobs AS (2007). Androgen deficiency, diabetes, and the metabolic syndrome in men. Curr Opin Endocrinol Diabetes Obes.

[CR16] Karazindiyanoğlu S, Cayan S (2008). The effect of testosterone therapy on lower urinary tract symptoms/bladder and sexual functions in men with symptomatic late-onset hypogonadism. Aging Male.

[CR17] La Vignera S, Calogero AE, D’Agata R, Di Mauro M, Tumino S, Condorelli R (2008). Testosterone therapy improves the clinical response to conventional treatment for male patients with metabolic syndrome associated to late onset hypogonadism. Minerva Endocrinol.

[CR18] Liu KE, Lo C-L, Hu Y-H (2014). Improvement of adequate use of warfarin for the elderly using decision tree-based approaches. Methods Inf Med.

[CR19] Miner MM, Seftel AD (2007). Testosterone and ageing: what have we learned since the Institute of Medicine report and what lies ahead?. Int J Clin Pract.

[CR20] Myon E, Martin N, Taïeb C, Heinemann LAJ (2009). Experiences with the French Aging Males’ Symptoms (AMS) scale. Aging Male.

[CR21] Quinlan JR (1987). Simplifying decision trees. Int J Man Mach Stud.

[CR22] Rosano GMC, Sheiban I, Massaro R, Pagnotta P, Marazzi G, Vitale C (2006). Low testosterone levels are associated with coronary artery disease in male patients with angina. Int J Impot Res.

[CR23] Tancredi A, Reginster J, Schleich F, Pire G, Maassen P, Luyckx F (2004). Interest of the androgen deficiency in aging males (ADAM) questionnaire for the identification of hypogonadism in elderly community-dwelling male volunteers. Eur J Endocrinol.

[CR24] Valenti G, Gontero P, Saccò M, Fontana F, Strollo F, Castellucci A (2009). Harmonized Italian version of the Aging Males’ Symptoms scale. Aging Male.

[CR25] Wang C, Nieschlag E, Swerdloff R, Behre HM, Hellstrom WJ, Gooren LJ (2008). ISA, ISSAM, EAU, EAA and ASA recommendations: investigation, treatment and monitoring of late-onset hypogonadism in males. Int J Impot Res.

[CR26] Wu FCW, Tajar A, Pye SR, Silman AJ, Finn JD, O’Neill TW (2008). Hypothalamic–pituitary–testicular axis disruptions in older men are differentially linked to age and modifiable risk factors: the European Male Aging Study. J Clin Endocrinol Metab.

[CR27] Wu FCW, Tajar A, Beynon JM, Pye SR, Silman AJ, Finn JD (2010). Identification of late-onset hypogonadism in middle-aged and elderly men. N Engl J Med.

